# Trustworthiness, Value, Danger, and Readability of ChatGPT-Generated Responses to Health Questions Related to Pulmonary Arterial Hypertension

**DOI:** 10.7759/cureus.71472

**Published:** 2024-10-14

**Authors:** Murat Kerkütlüoğlu, Erhan Kaya, Rasim Gökmen

**Affiliations:** 1 Cardiology, Kahramanmaraş Sütçü İmam University, Kahramanmaraş, TUR; 2 Public Health, Kahramanmaraş Sütçü İmam University, Kahramanmaraş, TUR

**Keywords:** artificial intelligence, chatbot, language models, patient information, pulmonary arterial hypertension, readability, safety

## Abstract

Aim: To enhance outcomes for patients with pulmonary arterial hypertension (PAH), comprehensive and individualized therapy is needed. A large language model called Generative Pre-trained Transformer (ChatGPT) has the ability to provide expert yet patient-friendly care. We wanted to determine how well ChatGPT could accurately and consistently respond to inquiries on knowledge and management for PAH.

Materials and methods: When 20 PAH patients were diagnosed, they were asked what concerns they had about PAH and what they had researched online. In the evaluation, it was determined that patients frequently searched the Internet for answers to eight queries. These eight queries were posed to ChatGPT, and their responses were recorded. Ten experts in the field of PAH assessed the trustworthiness, value, and hazard of the answers generated by the ChatGPT.

Results: According to evaluations conducted by experts, the ChatGPT-generated responses were deemed trustworthy with an average score of 8.4 (7.7-9.2) and valuable with an average score of 7.9 (7.4-8.2). Based on the statistical analysis, it can be inferred that most professionals believed that the utilization of prompts provided by ChatGPT did not present a substantial risk, with a mean of 2.1 (1.7-2.5). The answers were assessed for readability using two different indicators, namely the Flesch-Kincaid Grade Level (FKGL) and the Simple Measure of Gobbledygook (SMOG). The average FKGL value was determined to be 13.52 ± 2.40, indicating a "difficult" level of readability.

Conclusion: ChatGPT provides reliable PAH-related information, but it is important to seek professional medical advice before making any decisions regarding PAH. ChatGPT can only provide general information and support, but a qualified healthcare provider can offer tailored recommendations.

## Introduction

This article was previously presented as a meeting abstract at the 3rd International Congress on Innovative Approaches in Medical and Health Sciences on 08 October 2023.

The utilization of the internet for the purpose of obtaining health-related information is a prevalent occurrence. Patients frequently resort to seeking information online due to a lack of communication from their healthcare provider, either due to forgetfulness, inadequate explanation, or a failure to comprehend the information conveyed [1]. Frequently, patients engage in the act of researching and presenting information to their healthcare provider to verify or challenge the decisions made regarding their treatment [2]. The ChatGPT, which is a language model founded on the third generation of the Generative Pre-trained Transformer (GPT-3) architecture, was introduced in November 2022. In a matter of weeks, the recently developed artificial intelligence (AI) system garnered a user base of 100 million and received frequent coverage in popular media outlets. ChatGPT is a robust natural language processing model with the ability to comprehend and produce textual content. The abundance of information available through this resource renders it highly valuable for diverse purposes, including but not limited to language interpretation, condensation of text, and generation of written content. A commonly expressed critique of ChatGPT pertains to the potential inaccuracy of the generated texts, which may occasionally produce erroneous or fictitious content. Within the field of AI, a hallucination refers to a response generated by an AI system that exhibits a high degree of confidence, despite lacking sufficient justification from its training data [3]. The language model generates a text that lacks a basis in reality or empirical evidence.

ChatGPT is a communication tool that is commonly utilized by healthcare professionals and consumers [4]. Large language models, such as ChatGPT, are poised to significantly alter the manner in which patients seek information pertaining to their health status [5]. A recent review conducted an investigation into the efficacy of ChatGPT in the domains of healthcare education, research, and practice. The review has also brought to attention certain limitations that may be encountered [6]. One of the constraints identified in the study relates to the potential for erroneous information and hallucinatory effects. The review suggests that further research is necessary to assess the substance of language models as well as their potential to promote academic and scientific progress, with a specific emphasis on healthcare environments.

Patients diagnosed with pulmonary arterial hypertension (PAH) exhibit a positive attitude toward utilizing internet-based technologies [6]. Patients with PAH and their caregivers have the option to seek information pertaining to their condition on ChatGPT. However, the reliability, caliber, and readability of the information available on ChatGPT with regard to PAH remain uncertain. Despite the fact that ChatGPT is readily accessible, free, and simple to comprehend, these characteristics may not always apply to us. Can ChatGPT be considered a credible source of information pertaining to PAH?

## Materials and methods

When 20 PAH patients were diagnosed, they were asked what concerns they had about PAH and what they had researched online. In the evaluation, it was determined that patients frequently searched the internet for answers to eight queries. Table 1 contains the eight queries posed to ChatGPT. The ChatGPT-generated responses have been logged (Supplementary material 1).

**Table 1:** ChatGPT was asked eight questions about PAH from virtual patients PAH: pulmonary arterial hypertension

Questions	Percent
1. What is pulmonary arterial hypertension?	100% of patients
2. What causes pulmonary arterial hypertension?	100% of patients
3. Is pulmonary hypertension the same as hypertension as we know it?	60% of patients
4. What are the symptoms of pulmonary arterial hypertension?	100% of patients
5. Who has pulmonary arterial hypertension?	80% of patients
6. Pregnancy in a patient with pulmonary arterial hypertension?	60% of patients
7. How is pulmonary arterial hypertension diagnosed?	100% of patients
8. How is pulmonary arterial hypertension treated?	100% of patients

A questionnaire was created exclusively for the purpose of this research (Supplementary material 2). A set of questionnaires was administered to all 10 medical experts who are members of the Turkish Cardiology Association's PAH study group and are cardiologists actively involved in clinical research and studies in this field. Ten cardiologists were selected using the random sampling method from among the volunteer members of this study group. The ChatGPT responses were evaluated by these professionals based on trustworthiness, value, and danger, utilizing a numerical rating system ranging from 1 to 10. The greater the numerical value assigned to a response, the greater its perceived level of trustworthiness, value, or danger. This methodology has been used as explained in a previous study addressing a similar topic [1].

The readability of websites was assessed using the Flesch-Kincaid (FK) grade and the Simple Measure of Gobbledygook (SMOG). The scores from these two formulas were obtained using an online tool (https://www.webfx.com/tools/read-able/). Indicators such as FK and SMOG are formulated based on the word in the sentence and the number of syllables in the word. High scores indicate low readability levels of websites. The readability of the text was categorized as "easy" (grade 6), "average" (grades 6-10), or "difficult" (grade >10) based on the Flesch-Kincaid Grade Level (FKGL) indicator (Table 2) [7,8].

**Table 2:** Readability formulas provide indications of text readability

Parameters	Formulas
FKGL	(11.8 x (total syllables / total words)) + (0.39 x (total words / total sentences)) - 15.59
SMOG	3.1291 + 1.0430$&radic;((complex words) (30/sentences))$
FKGL: Flesch-Kincaid Grade Level; SMOG: Simple Measure of Gobbledygook	

Means and quartiles are reported for descriptive statistics. A line graph is used to graphically represent data.

This study received approval from the Ethics Committee of Kahramanmaraş Sütçü İmam University for Medical Research, under approval number 2023/15-02.

## Results

**Figure 1:**
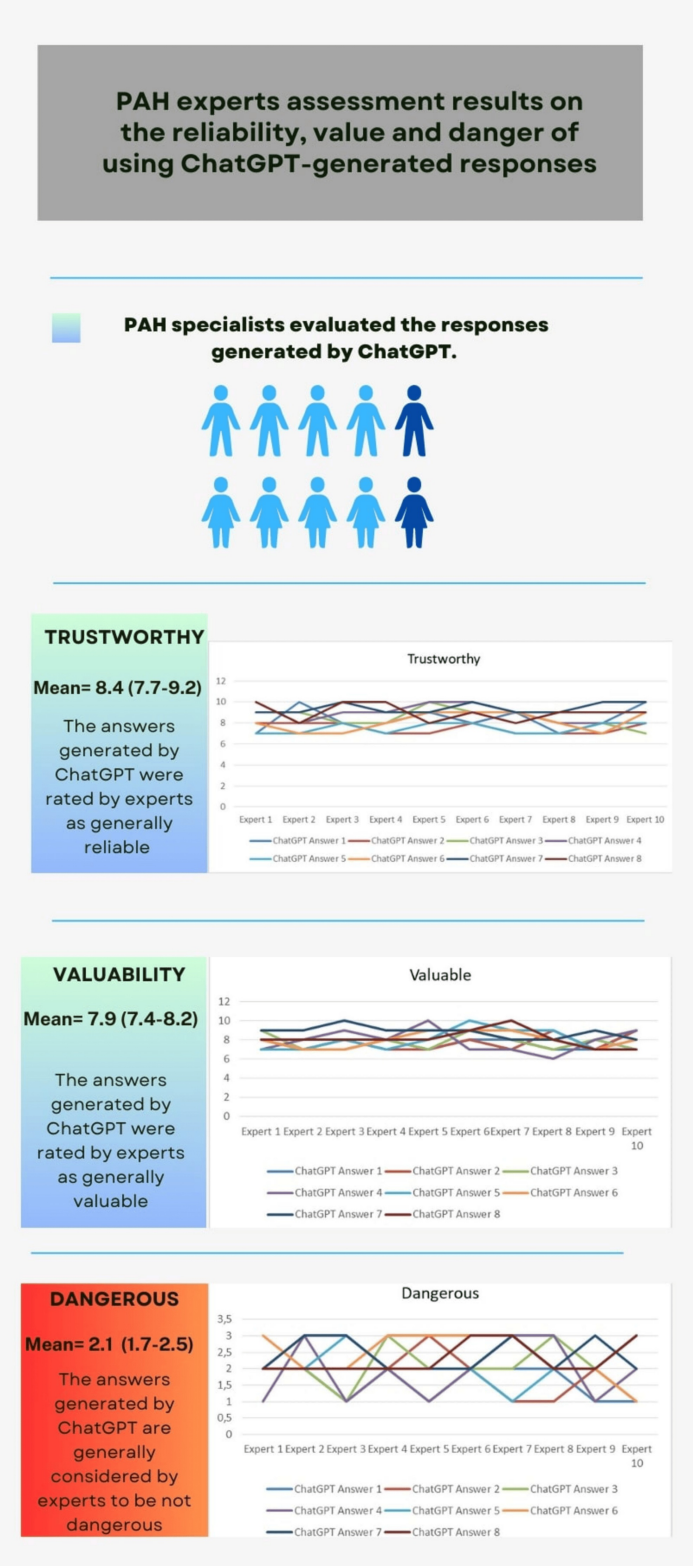
PAH experts assessment result on the reliability, value, and danger of using ChatGPT-generated responses PAH: pulmonary arterial hypertension

In the examination of ChatGPT's responses in terms of trustworthiness, value, and danger, responses from the experts' questionnaire determined that the responses produced by ChatGPT were trustworthy, with a mean score of 8.4 (7.7-9.2), and valuable, with a mean score of 7.9 (7.4-8.2). According to the statistical analysis, with a mean of 2.1 (1.7-2.5), the majority of professionals held the view that utilizing the prompts supplied by ChatGPT did not pose a significant risk (Figure 1).

According to a panel of medical expert cardiologists in the field, the findings generally show that ChatGPT has the capacity to produce trustworthy and significant data regarding PAH.

In their explanations, the experts expressed their appreciation for ChatGPT's nuanced and comprehensive responses, as well as their recommendation to consult with healthcare professionals for further direction.

The readability of the obtained ChatGPT responses was evaluated using the scores derived from the Flesch-Kincaid (FK) grade and the Simple Measure of Gobbledygook (SMOG) formulas, with average values found to be 13.52 for FKGL and 12.49 for SMOG (Table 3).

**Table 3:** Information quality scores and readability indicators assess the grade level of the content

	Mean (SD)	Median (min-max)
FKGL	13.52 (2.40)	13.10 (10.30-17.50)
SMOG	12.49 (2.27)	11.95 (9.70-16.10)
FKGL: Flesch-Kincaid Grade Level; SMOG: Simple Measure of Gobbledygook		

## Discussion

PAH is a chronic and severe cardiopulmonary disorder that is characterized by the proliferation of cells and fibrosis in the small pulmonary arteries. This leads to a gradual increase in pulmonary vascular resistance [9]. The development of PAH initiates in the pulmonary circulation; however, it is the primary cause of morbidity and mortality due to right heart failure [10]. Contemporary therapeutic approaches for PAH entail the intervention of aberrant signaling pathways in the pulmonary vasculature, with the objective of mitigating the afterload on the right ventricle and forestalling the advancement toward right-sided heart failure and mortality [9]. In the last two decades, there have been significant changes in the epidemiology and treatment of PAH. PAH is a disease that is not commonly found, with a projected occurrence of 15-50 cases per million people [11]. Individuals afflicted with uncommon diseases, such as PAH, exhibit a strong desire to obtain knowledge pertaining to their condition and pursue solutions to their inquiries regarding the prognosis of particular circumstances and therapeutic procedures through online resources.

Consumers may now easily access a wealth of highly technical information thanks to the development of the internet. Because of this, a significant number of individuals are now turning to Google rather than a qualified medical professional for the medical advice they need. While it doesn't appear that this practice will disappear any time soon, ChatGPT and other types of AI could displace it. The emergence of ChatGPT is attributed to the progress made in large language models, signifying a new era of AI technologies [12]. According to a report, ChatGPT reached 100 million users within 64 days of its launch on November 30, 2022 [13]. The platform is renowned for its proficiency in generating text that closely resembles human quality and covers a diverse array of subjects [13].

The term "artificial intelligence" refers to a cross-disciplinary field that combines computer science and linguistics with the goal of developing machines that possess the ability to execute tasks that typically necessitate human intelligence [14]. The aforementioned tasks encompass a range of cognitive skills, including but not limited to learning, adaptation, rationalization, comprehension of abstract concepts, and responsiveness to intricate human attributes such as attention, emotion, and creativity [15]. The primary purpose of the system was not to cater to healthcare needs, and its potential in addressing patient queries remains unexplored [16]. The reception of ChatGPT within the scientific community and academic circles has been varied, indicating the longstanding debate surrounding the advantages and drawbacks of sophisticated AI technologies [17-19]. ChatGPT has the potential to enhance conversational and writing abilities, thereby improving the effectiveness and precision of the desired outcome [6]. However, there have been concerns raised regarding potential bias stemming from the datasets utilized in the training of ChatGPT. This may restrict its abilities and lead to factual errors, which are disconcertingly scientifically feasible, a phenomenon known as hallucination [20].

Nonetheless, the comprehensive positive evaluation of dependability, as evidenced in our investigation assessing the responses proffered by ChatGPT concerning PAH, has similarly been corroborated in prior research endeavors exploring the precision of the responses produced by ChatGPT. Antaki et al. conducted a study to examine the precision of ChatGPT in the field of ophthalmology. The study employed a set of multiple-choice questions sourced from the Ophthalmic Knowledge Assessment Program [21]. The academic performance of ChatGPT was evaluated, and it was determined that the scores obtained on the exams were comparable to those of a typical first-year resident. The recorded percentages of 55.8% and 42.7% show that the results were noteworthy and promising [21]. Thus, this research has recognized the significance of patients' feedback. A further investigation was conducted to assess the precision and consistency of answers pertaining to knowledge, management, and emotional support regarding cirrhosis and hepatocellular carcinoma [22]. The study conducted by Duong and Solomon aimed to evaluate the efficacy of ChatGPT in addressing queries related to genetics in comparison to human participants [23]. The researchers arrived at the conclusion that ChatGPT offers prompt and precise answers to a diverse set of inquiries pertaining to genetics. They further noted that ChatGPT has the potential to facilitate the accessibility of information for laypersons [23]. In their research, Ziebland et al. claimed that severe illness frequently undermines people's perceptions of themselves as capable members of society [24]. Patients diagnosed with PAH can utilize AI technology to acquire knowledge and skills to showcase their proficiency in managing their condition amidst the challenges posed by the illness. Our study conducted on ChatGPT's responses and recommendations by experts in the field of PAH concluded that they were deemed secure, beneficial, and devoid of any potential hazards.

Patients with a low educational level may struggle to understand data that has a high readability score. In addition to the quality of the information, health-related content should also aim to present easily understandable material [8]. In our study, when examining the response texts using two different readability scores, we found that the average FKGL value was 13.52 and the average SMOG value was 12.49. In a study examining the readability of websites for ankylosing spondylitis, the average FK and SMOG scores were 8.59 and 7.33, respectively. In another study evaluating the readability of websites for plantar fasciitis and calcaneal spur, the average scores were 7.27 for FKGL and 6.89 for SMOG [7,8]. This situation may make it more challenging for the general population to obtain information from ChatGPT compared to websites and could limit the information they receive.

This study has some limitations. First and foremost, in the stage of determining the questions, starting from the problems that the patients are curious about, although it enables the identification of a patient-oriented problem, it causes the identification to be subjective. Different research questions could have been based on different groups. The second is related to the fact that AI applications respond differently to questions at different times. Since this appearance may potentially jeopardize the reproducibility of the information, we kept the answer samples as images. Moreover, there are differences between the evaluators who check the accuracy of the information. In our methodology, there is no defined grading rubric or systematic approach for the evaluation process of ChatGPT responses; the ratings are based on the subjective assessments of medical experts actively working in this field. For this reason, we included a large number of observers' opinions.

## Conclusions

In conclusion, ChatGPT demonstrates extensive expertise in the realm of healthcare, attributable to the utilization of expansive datasets during its training process. The veracity of the data furnished by ChatGPT is contingent upon an accurate comprehension and response to the inquiries posed. The reliability of health-related information furnished by ChatGPT is widely acknowledged by professionals in the field. Nonetheless, a precise diagnosis or treatment suggestion for any health issue necessitates an evaluation of the particular circumstances and a personalized methodology. Hence, it is not reasonable to anticipate that the data furnished by ChatGPT would serve as a substitute for the information dispensed by a medical practitioner or an expert in the field.

## Figures and Tables

**Figure 2:**
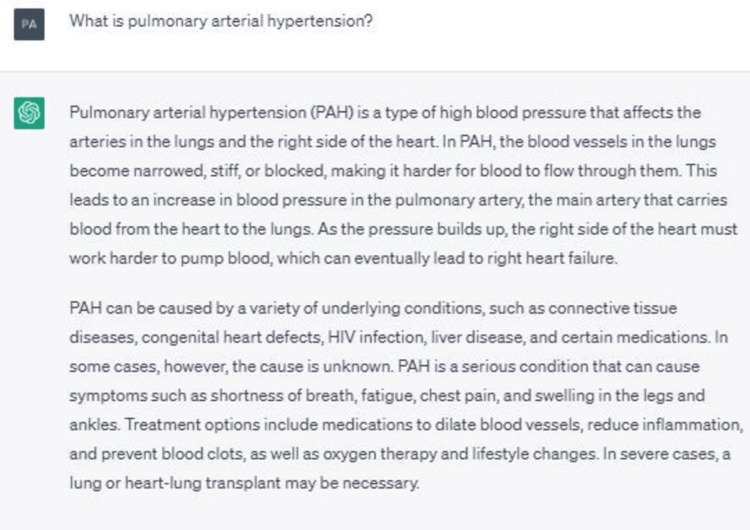
Question 1

**Figure 3:**
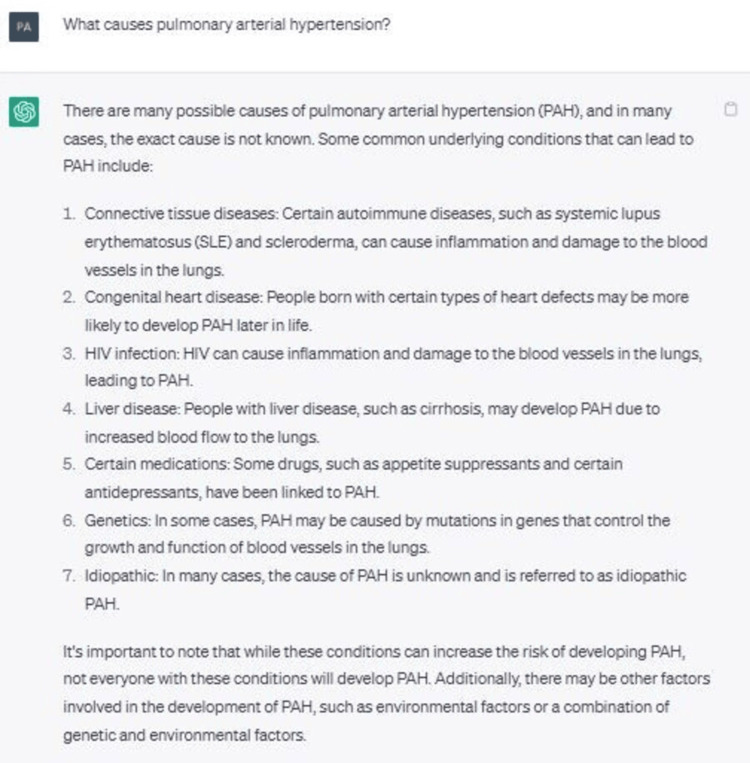
Question 2

**Figure 4:**
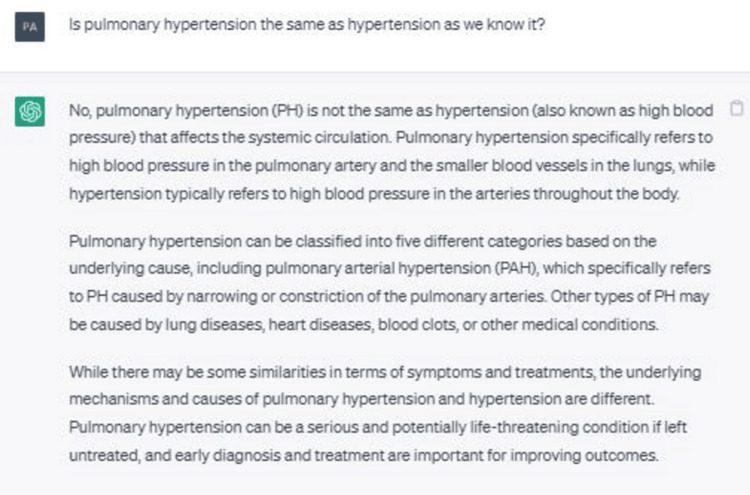
Question 3

**Figure 5:**
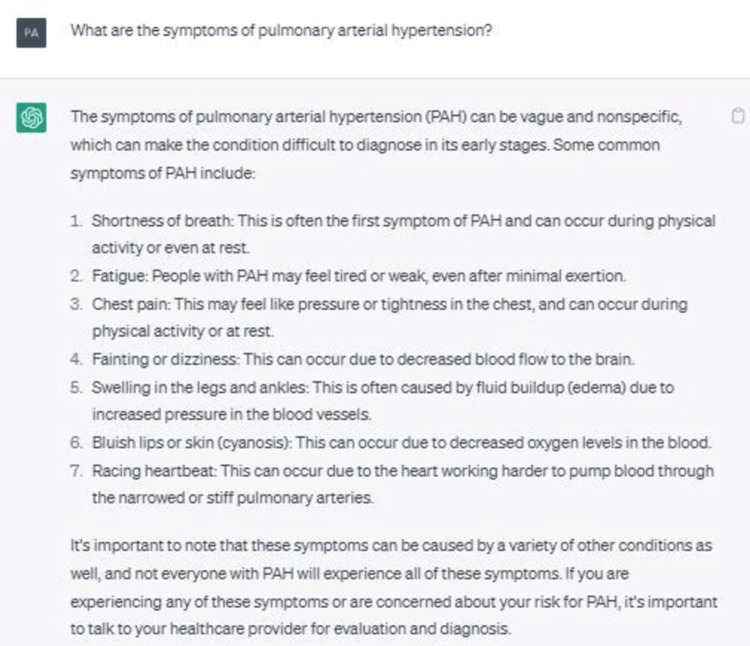
Question 4

**Figure 6:**
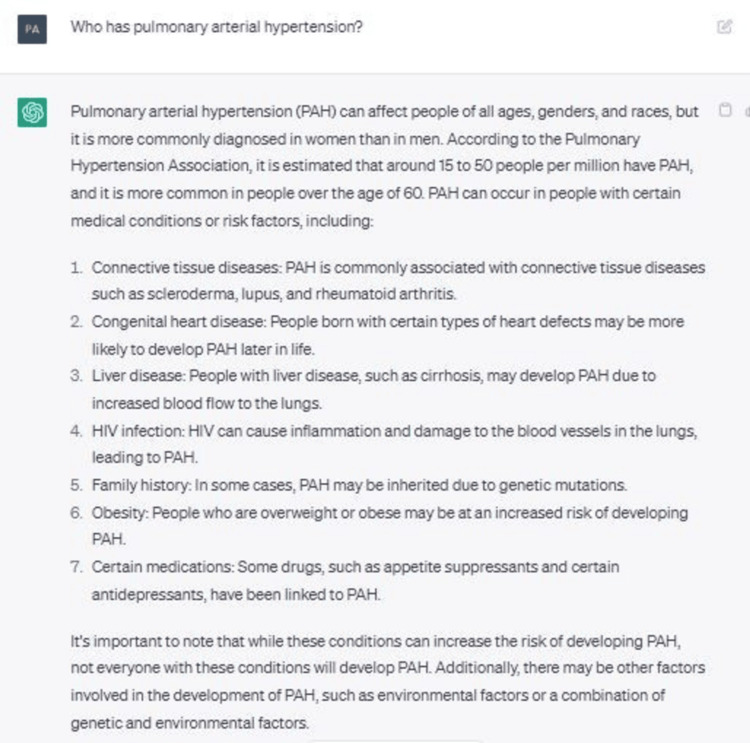
Question 5

**Figure 7:**
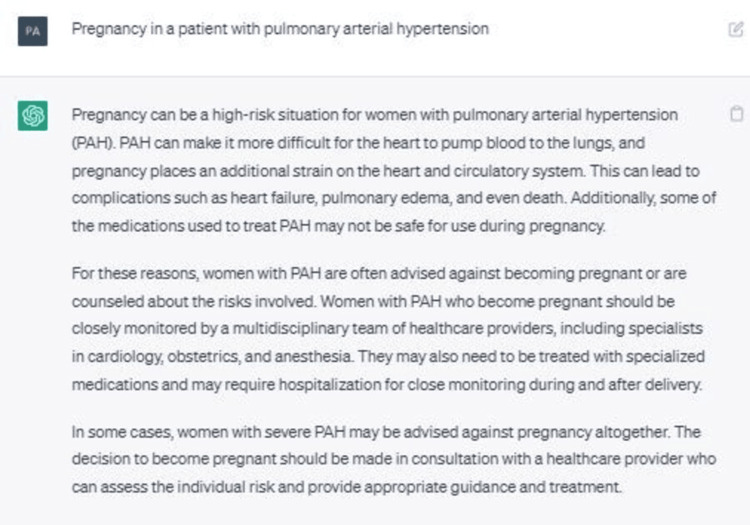
Question 6

**Figure 8:**
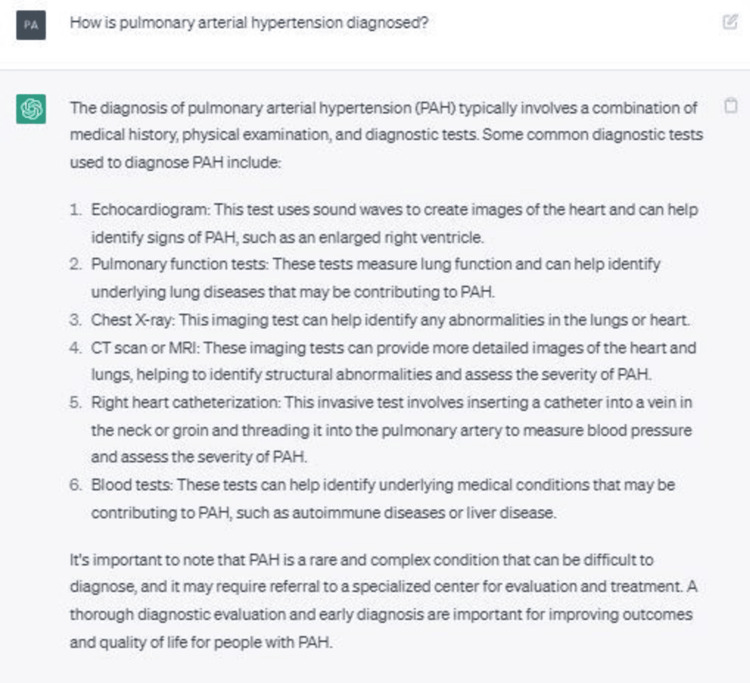
Question 7

**Figure 9:**
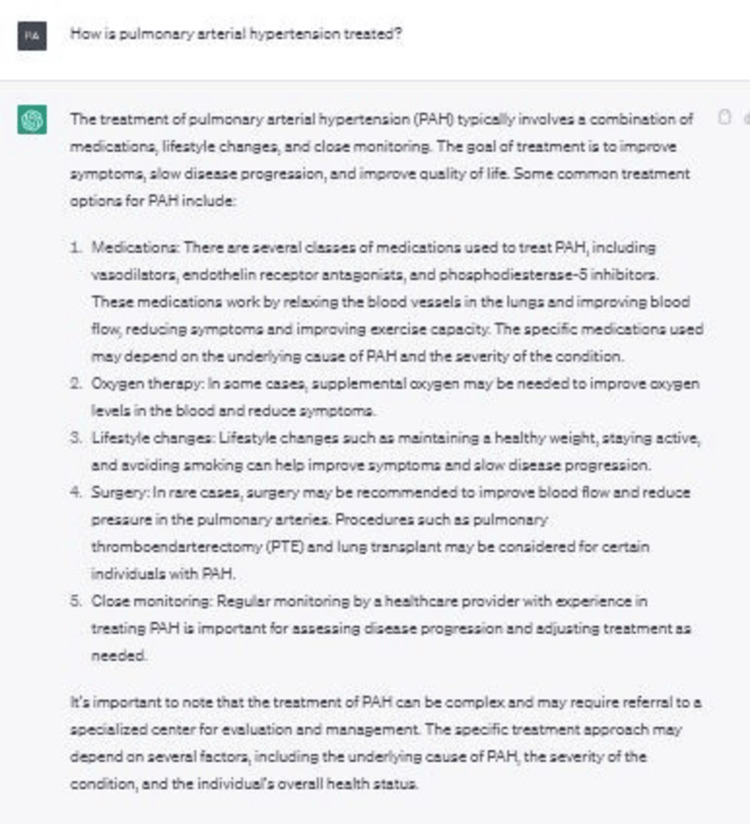
Question 8

**Figure 10:**
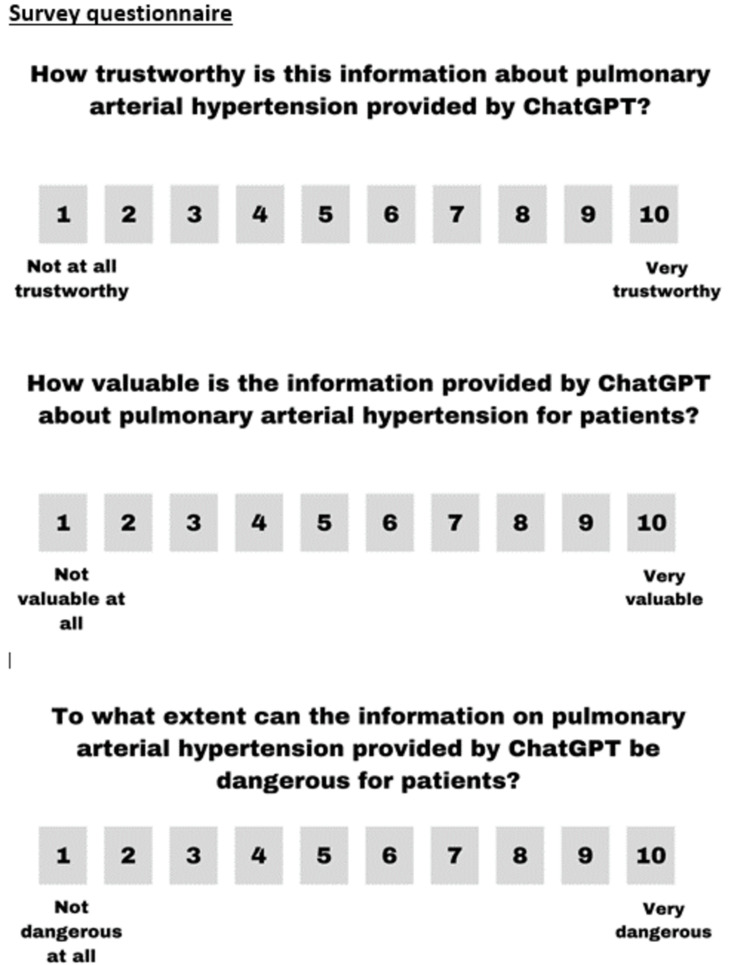
Survey questionnaire

## Appendices

Supplementary material 1

Supplementary material 2
